# The Association between Weather Conditions and Admissions to the Paediatric Intensive Care Unit for Respiratory Syncytial Virus Bronchiolitis

**DOI:** 10.3390/pathogens10050567

**Published:** 2021-05-07

**Authors:** Rosalie S. Linssen, Bibiche den Hollander, Louis Bont, Job B. M. van Woensel, Reinout A. Bem, on behalf of the PICE Study Group

**Affiliations:** 1 Paediatric Intensive Care Unit, Emma Children’s Hospital, Amsterdam University Medical Centers, Location AMC, 1105 AZ Amsterdam, The Netherlands; b.denhollander@amsterdamumc.nl (B.d.H.); j.b.vanwoensel@amsterdamumc.nl (J.B.M.v.W.); r.a.bem@amsterdamumc.nl (R.A.B.); 2 Amsterdam Reproduction & Development (AR&D) and the Amsterdam Infection & Immunity (AR&I) Research Institutes Medical Centers, Location AMC, 1105 AZ Amsterdam, The Netherlands; 3 UMCU Laboratory of Translational Immunology, University Medical Center Utrecht, Wilhelmina Children’s Hospital, 3584 EA Utrecht, The Netherlands; l.bont@umcutrecht.nl; 4 Respiratory Syncytial Virus Network (ReSViNET) Foundation, 3703 CD Zeist, The Netherlands

**Keywords:** bronchiolitis, respiratory syncytial virus, paediatric critical care, epidemiology, weather, meteorological, climate

## Abstract

Respiratory syncytial virus (RSV) bronchiolitis is a leading cause of global child morbidity and mortality. Every year, seasonal RSV outbreaks put high pressure on paediatric intensive care units (PICUs) worldwide, including in the Netherlands, and this burden appears to be increasing. Weather conditions have a strong influence on RSV activity, and climate change has been proposed as a potential important determinant of future RSV-related health care utilisation. In this national study spanning a total of 13 years with 2161 PICU admissions for RSV bronchiolitis, we aimed (1) to identify meteorological variables that were associated with the number of PICU admissions for RSV bronchiolitis in the Netherlands and (2) to determine if longitudinal changes in these variables occurred over time as a possible explanation for the observed increase in PICU burden. Poisson regression modelling was used to identify weather variables (aggregated in months and weeks) that predicted PICU admissions, and linear regression analysis was used to assess changes in the weather over time. Maximum temperature and global radiation best predicted PICU admissions, with global radiation showing the most stable strength of effect in both month and week data. However, we did not observe a significant change in these weather variables over the 13-year time period. Based on our study, we could not identify changing weather conditions as a potential contributing factor to the increased RSV-related PICU burden in the Netherlands.

## 1. Introduction

**Figure 1:**
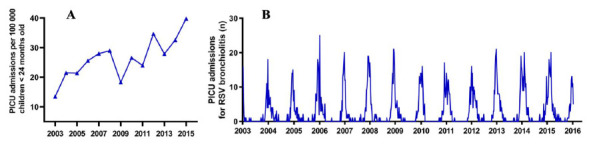
PICU admissions for RSV bronchiolitis from 2003 to 2016 in the Netherlands. (**A**) line represents the total number of PICU admissions for RSV bronchiolitis per 100,000 children in the population <24 months old (adapted with permission from [6]). (**B**) line presents the distribution of the number of PICU admissions for RSV bronchiolitis per week over the study period. Note: on the *x*-axis, the years refer to RSV seasons 2003 to 2016. As RSV infections and subsequent RSV bronchiolitis PICU admissions peak during the winter, data were shifted into ‘seasons’ from 1 July up to and including 30 June in the year thereafter [6].

Respiratory syncytial virus (RSV) bronchiolitis is a major cause of infant morbidity and mortality worldwide [1,2]. Up to 5% of RSV-infected children experience acute deterioration of bronchiolitis, necessitating admission to a paediatric intensive care unit (PICU) for mechanical ventilation [3]. Over the last two decades, studies from various countries around the world have reported an increase in the number of PICU admissions for RSV bronchiolitis, associated with rising health care costs and increased pressure on health care systems [3,4,5]. Likewise, the number of PICU admissions for RSV bronchiolitis has increased approximately fourfold in the Netherlands between 2003 and 2016 (Figure 1A) [6], but the reasons for this increase remain to be elucidated. Further knowledge on the possible causes driving this increased RSV-related burden is warranted to allow for optimal future strategic planning of PICU capacity.

There is a clear relationship between the RSV load among the population and local climates, as derived from RSV surveillance and/or hospital admission data [7,8,9,10,11]. While in temperate climates, RSV activity peaks during the winter following decreasing temperatures, it shows a more continuous pattern in tropical regions with peaks in the rainy season, with precipitation and humidity being important meteorological determinants [7,8,9,11,12,13]. However, in the current era of climate change, local shifts in the seasonal pattern of RSV outbreaks have been predicted through the dynamic modelling of weather conditions [8]. Such shifting in the seasonal pattern of RSV outbreaks may subsequently affect the RSV-related PICU burden.

Despite this well-established relationship between the weather and seasonal patterns of RSV transmission, we have only limited knowledge about meteorological conditions in relation to acute clinical deterioration in children with RSV bronchiolitis necessitating PICU admission. Changes in meteorological variables can have a direct impact on the local microenvironment in the airways, including mucociliary clearance and airway smooth muscle cell contraction [14,15,16,17,18]. Unsurprisingly, fluctuations in air pressure, humidity, solar radiation, temperature, and wind speed have been associated with exacerbations of a number of respiratory diseases, including chronic obstructive pulmonary disease (COPD) and asthma [19,20,21,22]. In addition, meteorological conditions such as radiation (e.g., solar UV light) may impact viral stability and possibly the patient’s viral load [23,24], thereby influencing the risk of severe RSV bronchiolitis and PICU admission [25].

While studying the increased PICU admissions for RSV bronchiolitis over the last two decades [6], we observed an interesting change in the pattern of PICU admissions for RSV bronchiolitis over time: instead of higher peaks in the PICU admissions (e.g., an increase in the maximum amount of PICU admissions in RSV endemic weeks), we observed that the PICU admissions for RSV bronchiolitis were spread out over a longer period of time (e.g., a ‘broadening of the seasonal curve’) (Figure 1B). Although the pattern in PICU admissions for RSV bronchiolitis still remained highly seasonal, this pattern somewhat appeared to shift into a more continuous pattern as known from tropical climates. However, as the RSV load among the population remained stable during this period [6], this led us to hypothesize that local weather conditions may influence the risk of PICU admission for RSV bronchiolitis, and that this may be subject to change over time.

This study aims to (1) identify weather variables that are associated with PICU admissions for RSV bronchiolitis in the Netherlands, and (2) to determine if these variables have changed over time as a possible explanation for the observed increase in PICU burden of RSV bronchiolitis in the Netherlands between 2003 and 2016. These data are needed to inform public health epidemiologists and PICU clinicians involved in future strategic paediatric critical care resource planning.

## 2. Results

During the study period, 2161 children up to 24 months old were admitted to a PICU in the Netherlands with a confirmed case of RSV bronchiolitis. Population-based estimates on the number of PICU admissions for RSV bronchiolitis per 100,000 children <24 months old in the population showed a fourfold increase: from 13.5 per 100,000 children in 2003 to 48.0 per 100,000 children in 2016 [6] (Figure 1A). In absolute admission numbers, this study reported an increase of 166 annual RSV bronchiolitis-related PICU admissions when compared with the years 2003 (83 admissions) to 2016 (249 admissions) [6]. During the same period, no increase could be observed in RSV isolations among the general population (data provided by the Dutch Society for Medical Microbiology, RIVM) [6]. Detailed information on patient characteristics, comorbidity, and treatment as well as the increase of PICU admissions related to the RSV activity among the population over time has been described elsewhere [6].

### 2.1. Association between Weather Variables and PICU Admissions

As expected, based on the RSV activity pattern among the population in the Netherlands, PICU admissions for RSV bronchiolitis followed a strong seasonal (winter) pattern (Pearson chi-square 79,352, df 43, *p* < 0.01). To study if any correlations between weather variables and PICU admissions could be detected, we first carried out Poisson regression for all different meteorological variables as a predictor separately, using data from all months of the year (Table 1). Explorative factor analysis (see Methods and Appendix A) indicated three groups of meteorological conditions containing variables that showed high correlation among each other (and thus multicollinearity): (1) radiation variables (cloud cover, humidity, % of longest sunshine duration, sunshine radiation, and global radiation), (2) temperature variables (minimum temperature, mean temperature, and maximum temperature), and (3) the remaining variables (wind speed and precipitation) (Box 1). For the data aggregated per month (whole year), the variables global radiation and maximum temperature best predicted the number of PICU admissions for RSV bronchiolitis. Less global radiation and a lower maximum temperature resulted in more PICU admissions (Table 1). Both wind speed and precipitation provided only a little additive value in the prediction of PICU admissions (Table 1). We therefore chose to create a combined model consisting only of maximum temperature and global radiation. A combined model of these two variables predicted the RSV bronchiolitis PICU admissions the best (*p* < 0.001) for all months.

Box 1 **Definitions.**

**                      A. Meteorological variables**

**Cloud cover**

Determination of the coverage of the sky through visual measurements or visibility meters. Determined hourly and reports to what extend the sky is clear (category 0) to fully covered with clouds (invisible sky, category 9) while making use of eight categories (octants). For each day the mean cloud cover is determined based on all hourly observations.

**Relative humidity**

Relative humidity (%) is the volume of water vapour in the air at a certain temperature divided by the maximum volume of water that can be air-contained at that same temperature.

**Sunshine duration & % of maximum possible sunshine duration**

The sunshine duration (in 0.1 h) is automatically calculated through an algorithm that uses the 10-min radiation measurements of that day. The % of maximum sunshine duration (%) is the sunshine duration divided by the longest possible sunshine duration in hours for that specific day. As such, it takes into account the seasonal variation in the length of days in the Northern Hemisphere.

**Global radiation**

Global radiation: the sum of the direct radiation from the sun (which passes directly to the earth’s surface) and the diffuse radiation spread by particles in the atmosphere or reflected by clouds. As such, the global radiation includes visible light, infrared and ultraviolet radiation (UVA and UVB). Reported in (J/cm^2^).

**Temperature**

The minimum, mean temperature and maximum temperature in 0.1 °C.

**Wind speed**

Horizontal movement of air at a height of 10 m above the ground (reference surface). Mean wind speed refers to the mean of 24-hourly wind speed measurements. Reported in m/s.

**Precipitation**

The total amount of precipitation per day (rain, snow etc) measured by a rain gauge and measured in mm.

**                          B. Time lags**

**Time lag:** the period of time between two events (weather and PICU admission). To take a possible delayed effect in a certain relationship into account, we studied this relationship using different lags (e.g., 7 and 14 days).

To take the strong seasonal pattern of RSV activity in the Netherlands into account, we studied the effect of the different weather variables on PICU admissions, including data from only the months falling within the RSV season (September–April) (Table 1). Again, maximum temperature and global radiation showed the highest strength of effect, and a model that combined these variables best predicted PICU admissions for RSV bronchiolitis (*p* < 0.001).

**Figure 2:**
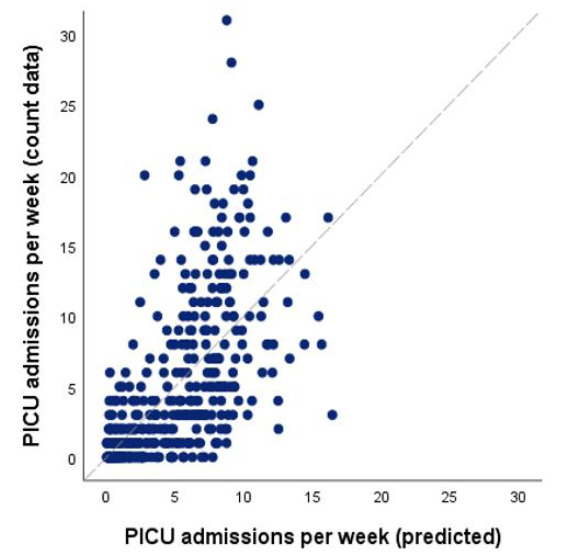
Model performance for Poisson regression modelling with maximum temperature and global radiation included in the model as a predictor for the number of PICU admissions for RSV bronchiolitis, using weekly data for only the RSV season (*p* < 0.001). *x*-axis: expected admissions counts per week based on the Poisson regression analysis. *y*-axis: observed admission counts per week. In a perfectly performing model all data points would follow exactly a 45° diagonal line.

Even so, when aggregating data per week to explore a potentially more direct influence of weather variables on PICU admissions, the following was observed: maximum temperature and global radiation showed the strongest effect (Table 1), and combining these variables best predicted PICU admissions for RSV bronchiolitis. Repeated analysis with a time lag of seven days (Box 1) also identified global radiation (*p* < 0.001) and maximum temperature (*p* < 0.001) as the best predicting variables but not did improve the predictions on PICU admissions compared to the weekly model in which no time lag was used. Figure 2 presents the expected PICU admissions based on this combined model for the RSV endemic weeks (*p* < 0.001) compared to the count data without a time lag (observed number of admissions).

As can be observed from these different analyses in Table 1, the strength of effect for maximum temperature was lower in a model that used weekly aggregated data from only within the RSV season as compared to a model that included data from the whole year. This can be expected from the seasonal RSV pattern. Interestingly, however, global radiation showed a relatively stable strength of predictive effect on PICU admissions for RSV bronchiolitis when using both monthly and weekly aggregated data (Table 1).

### 2.2. Changes in Weather Variables over Time

To assess for longitudinal changes in the Dutch weather over the study period, we carried out linear regression analysis over time for all weather variables (Table 2). We paid specific attention to global radiation and maximum temperature, as these were the variables that showed the strongest predictive effect on PICU admissions (see above). Over the study period (2003 to 2016), only cloud coverage (β 0.18; SE 0.0) and wind speed (β 0.09; SE 0.00) increased significantly. When solely assessing the weather during RSV seasons, only cloud coverage significantly changed between 2003 and 2016 (β 0.16; SE 0.0).

## 3. Discussion

This study assessed the relationship between meteorological variables and the number of PICU admissions for RSV bronchiolitis over a 13-year time period in the Netherlands. The number of PICU admissions for RSV bronchiolitis was best predicted by a model that combined maximum temperature and global radiation, which was also true when focusing only on data within the RSV season (winter). We did not observe a significant change over time in these two weather variables during the study period.

As for many countries with temperate climates, the seasonal pattern of the RSV infection rate in the Netherlands is well established [11]. Multiple factors that may possibly contribute to the seasonality of RSV infections have been identified, such as behavioural changes leading to indoor crowding, possibly in combination with increased time spent indoors at day cares [11,26,27,28], decreased resistance to infection due to low vitamin D levels [29,30,31], and meteorological factors directly influencing RSV particle stability and survival [32,33].

In a previous study from the Netherlands, which analysed surveillance data covering an 8-year period, relative humidity, temperature, and cloud coverage showed good correlation with RSV activity among the population [11]. Our study partly confirms these findings. Yet, global radiation, a meteorological variable which is affected by humidity and cloud coverage and which showed collinearity with these two variables in our explorative factor analysis, was found to be a better predictor of PICU admissions for RSV bronchiolitis in our dataset.

Although it is evident that PICU admissions will predominantly fall within the RSV season, RSV activity data from surveillance laboratories in relation to weather conditions cannot simply be extrapolated to the burden of PICU admissions. For example, it is possible that specific weather conditions contribute more directly to the severity of RSV bronchiolitis and are thus associated with an increased risk of PICU admission. In this light, the relationship between PICU admissions and the meteorological variables of temperature and radiation observed in this study may be interesting.

Lower temperature has been identified as a predominant factor in explaining RSV outbreaks [8,9,11,34,35] but has also been associated with the worsening of clinical symptoms and exacerbations of several respiratory diseases, such as asthma and COPD [14,15,16,19,22,36]. Possible mechanisms that may play a role in acute clinical deterioration in RSV bronchiolitis could include bronchoconstriction [14,15,16] and hyperventilation due to lower temperature, leading to increased evaporation of airway surface fluid [37,38]. Additionally, the inspiration of cold air may also have a direct impact on the mucociliary clearance through impaired cilia beating [17,18], increasing the risk of airway obstruction by mucus or causing an impairment of the immune responses of macrophages and granulocytes in the airways [39]. In this study, global radiation showed a stable strength of effect in predicting PICU admissions for both the monthly and weekly data. This may suggest a more direct relationship between the level of radiation and the risk for PICU admission. A previous study with RSV surveillance data also observed UV light to be inversely correlated to RSV activity [9]. Hypothetically, the relationship between PICU admissions and global radiation may be relevant, as increased radiation (e.g., UV and infrared light) is known to decrease virus particle stability and replication [23,40]. As such, reduced radiation in combination with colder temperature may allow for better stability of virions inside respiratory droplets, and this may increase viral load in patients [25,41,42,43,44,45]. Importantly, a relationship between certain meteorological variables and virulence may exist not only for RSV but also for other respiratory viruses. For example, for influenza, an important pathogen for both adults and children that shows seasonal outbreaks in temperate climates [46], a relationship between temperature, humidity, and viral stability has also been described [47].

A dynamic modelling study of the epidemics and outbreaks of RSV in relation to climate change indicated that changes in temperature-driven humidity and rainfall could drive seasonal RSV activity patterns towards a more continuous epidemic [8]. However, in our study, we could not find evidence that longitudinal changes in the meteorological variables that best predicted PICU admission (e.g., maximum temperature and global radiation) have contributed to the increase in PICU admissions for RSV bronchiolitis in the Netherlands between 2003 and 2016. Future studies stretching over longer time periods are needed to follow up on these data.

### Limitations

First, this study assesses the relationship between weather conditions and the number of PICU admission for RSV bronchiolitis. As we analysed data from the whole year and from within the RSV winter season only, by both month and week, we can allude to potential direct influences of these meteorological variables on the risk of PICU admission but, of course, cannot fully separate this from the more general effect of the weather on RSV activity among the population [29]. Although acute deterioration of RSV bronchiolitis in general occurs very rapidly, this limitation is especially important to realize since we have no information on the duration of symptoms and the length of hospital stay before PICU admission, and thus on the impact of indoor climate (e.g., temperature). Last, even though the time span of the data presented in this study comprised 13 years, which is in line with a previous study on the influence of climate change on RSV epidemics [8], this may be too short to draw strong conclusions on the effects of longitudinal changes of weather on the risk of PICU admissions for RSV bronchiolitis.

## 4. Materials and Methods

### 4.1. Collection of Patient Data

We collected data from children aged up to 24 months old admitted to a PICU in the Netherlands for a confirmed case of RSV bronchiolitis from 2003 to 2016, as described before [6]. In short, children were identified through the multicentre national PICU registry, the Dutch paediatric intensive care evaluation (PICE, https://www.pice.nl, accessed on 2 March 2018). As paediatric critical care in the Netherlands is exclusively provided in university medical centres, the PICE registry covers the full national PICU case load. Patients were eligible if they were coded in the PICE database under the Australian and New Zealand Paediatric Intensive Care Registry (ANZPIC) diagnosis as ‘bronchiolitis’ or ‘respiratory syncytial virus’. Subsequently, the individual medical records of all identified patients at all PICUs were checked manually for the correct diagnosis. Patients ≤ 24 months of age with a proven RSV infection, as detected with a rapid antigen test or PCR and reported in either the letter of admission, PICU record, or virology reports, and presenting with the constellation of clinical symptoms typical for bronchiolitis according to the American Academy of Paediatrics (AAP, 2006) or central apnoea’s were included in the study [48]. The number of PICU admissions for a confirmed case of RSV bronchiolitis were aggregated into weeks and months.

### 4.2. Collection of Meteorological Data

Meteorological data were obtained from the Royal Dutch Meteorological Institute website (www.knmi.nl, accessed on 7 November 2017). In line with a previous Dutch study [11], we use data derived from the Dutch main weather station ‘De Bilt’, which is located centrally in the Netherlands. In the Netherlands, distances are relatively small and hardly any differences in altitude exist (total surface area 41.540 km^2^). Data concerning the weather in the Dutch Caribbean were excluded from this study. We collected data on 10 variables (Box 1): cloud cover (octants, ranging from 0 for a clear sky and 9 for an invisible sky), mean relative humidity (%), maximum possible sunshine duration (%), sunshine duration (in 0.1 h)**,** global radiation (J/cm^2^), minimum temperature (in 0.1 °C), mean temperature (in 0.1 °C), maximum temperature (in 0.1 °C), mean wind speed (in m/s), and precipitation (in mm) per day. For the variables of minimum and maximum temperature, the lowest or the highest measurement were selected for each week, respectively. Temperature values provided in fractions were converted to whole degrees Celsius. When the precipitation was less than 0.05 mm, this was scored as a day without precipitation.

### 4.3. Data Processing and Analysis

Data were aggregated into means per week and month. Weeks were numbered according to the International Organisation for Standardization (ISO). The RSV endemic period in the Netherlands falls roughly between ISO week 35 to ISO week 18 of the following year, and we defined this period as the RSV season [7]. The incubation period of RSV ranges up to 7 days [1], and there might be a delay in the effect of the meteorological variables on the onset of disease or on clinical deterioration necessitating PICU admission. Therefore, we additionally analysed weekly data with a time lag (time delay) of 7 days (lag 7).

We tested for a seasonal pattern of PICU admissions using month data with a chi-square test. To study the relationship between individual meteorological variables and PICU admissions within the RSV season, we assessed for a correlation through bivariate correlation analysis. Then, we used explorative factor analysis (principal component analysis using varimax rotation with Kaiser normalization) to identify groups of meteorological variables that shared high correlation. To avoid multicollinearity, we did not combine multiple variables from within the same groups in our model. PICU admission data are ‘count data’ and not normally distributed. Therefore, Poisson regression was performed using a generalized linear model for all the different variables separately, and a multivariable model was created with the variables from each group that best predicted the outcome variable. The outcome variable was the number of PICU admissions for RSV bronchiolitis per week or per month. To determine which combination of variables best predicted PICU admissions (fit of the model), LR Pearson chi-square values (likelihood ratio test) were used. We also repeated statistical analyses with data only from within an RSV season, thus taking the seasonality of RSV activity into account. In addition, Poisson analyses were run both with monthly and weekly aggregated data to detect for more direct influences of the weather conditions on the number of PICU admissions. The strength of effect for each different weather variable with respect to the outcome variable (PICU admission) is presented by the betas.

To detect longitudinal changes within the different weather variables during the period under study (e.g., by climate change), we performed bivariate linear regression modelling over time. The betas from the linear regression model represent the slope of the estimated regression line and thus indicate the increase or decrease of that variable per season over the study period. Weather variables were checked for a normal distribution of data points.

All statistical analyses were performed using IBM SPSS Statistics 20. Significance was concluded when *p* < 0.05.

## 5. Conclusions

In this 13-year study on PICU admissions for RSV bronchiolitis in the Netherlands, we identified maximum temperature and global radiation as the weather variables having the strongest predictive effect on PICU admission burden. We did not observe any significant changes in these weather variables over time that could help explain the increase in PICU admissions for RSV bronchiolitis in the Netherlands.

## Figures and Tables

**Table 1:** Poisson regression analysis for the relationship between weather variables and number of PICU admissions for RSV bronchiolitis.

Variable	Month Data (All Months)		Month Data (RSV Season)		Week Data Lag 0 (RSV Season)		Week Data Lag 7 (RSV Season)	
	(β, SE)	LR test	(β, SE)	LR Test	(β, SE)	LR test	(β, SE)	LR Test
	Group 1							
Cloud coverage (octants)	98.95, 2.99	1338.29 *	72.39, 3.01	714.30 *	35.62, 1.91	407.59 *	39.29, 1.97	475.74 *
Relative humidity (%)	18.31, 0.51	1770.18 *	13.37, 0.57	709.90 *	8.54, 0.41	521.72 *	9.85, 0.42	656.11 *
% longest sunshine duration (%)	−8.60, 0.23	1638.96 *	−6.22, 0.23	835.17 *	−3.00, 0.15	454.44 *	−3.38, 0.15	554.51 *
Sunshine duration (0.1 h)	−6.45, 0.16	2584.62 *	−5.45, 0.18	1262.54 *	−3.60, 0.13	954.97 *	−3.99, 0.14	1103.63 *
Global radiation (J/cm^2^)	−0.29, 0.01	2987.52 *	−0.29, 0.01	1542.92 *	−0.27, 0.01	1601.56 *	−0.30, 0.01	1790.74 *
	Group 2							
Minimum Temperature (°C)	−2.43, 0.06	2162.93 *	−1.95, 0.07	871.50 *	−1.15, 0.05	593.14 *	−1.06, 0.05	492.39 *
Mean Temperature (°C)	−2.36, 0.05	2600.07 *	−2.05, 0.06	1212.85 *	−1.37, 0.05	941.36 *	−1.28 0.05	820.03 *
Maximum Temperature (°C)	−2.15, 0.05	2826.94 *	−1.92, 0.06	1417.15 *	−1.39, 0.04	1188.86 *	−1.32, 0.04	1070.82 *
	Group 3							
Wind speed (m/s)	9.66, 0.30	934.04 *	6.30, 0.32	369.06 *	3.06, 0.20	212.00 *	2.47, 0.21	133.32 *
Precipitation (mm)	−0.62, 0.18	12.58 *	0.67, 0.20	11.34 *	0.29, 0.09	8.94	0.12, 0.10	1.39

Legend: Variables were divided by 100 to obtain β and SE values in the tables. β: beta-coefficient; SE: standard error; LR: likelihood-ratio; * *p* < 0.001.

**Table 2:** Linear regression for all the different meteorological variables over time (2003–2016).

	All Weeks	RSV Season (Weeks 35–18)
Cloud coverage (octants)	β 0.18; SE 0.0, *p* < 0.01	β 0.16; SE 0.0, *p* < 0.01
Relative Humidity (%)	β −0.1, SE 0.0, *p* = 0.78	β −0.03, SE 0.0, *p* = 0.52
% Longest sunshine duration (%)	β −0.02, SE 0.00, *p* = 0.58	β −0.02, SE 0.01, *p* = 0.74
Sunshine duration (0.1 h)	β −0.02 SE 0.01 *p* = 0.55	β −0.02 SE 0.01 *p* = 0.73
Global radiation (J/cm^2^)	β −0.2, SE 0.12, *p* = 0.69	β −0.01, SE 0.15, *p* = 0.89
Minimum temperature (°C)	β 0.05 SE 0.01 *p* = 0.18	β 0.06 SE 0.01 *p* = 0.19
Mean temperature (°C)	β 0.02, SE 0.01 *p* = 0.55	β 0.04, SE 0.02 *p* = 0.39
Maximum temperature (°C)	β −0.001, SE 0.01, *p* = 0.98	β −0.01, SE 0.02, *p* = 0.70
Wind speed (m/s)	β 0.09, SE 0.00, *p* = 0.01	β 0.08, SE 0.00, *p* = 0.09
Precipitation (mm)	β 0.04, SE 0.00, *p* = 0.32	β 0.03, SE 0.01, *p* = 0.51

Legend: β: beta-coefficient; SE: standard error.

## Data Availability

Restrictions apply to the availability of these data. Data was obtained via the PICE study group and are available from the authors with the permission of the PICE study group.

## Appendix A

**Table A1:** Factor analysis (Rotated Component Matrix). Rotation method: Varimax rotation with Kaiser Normalization.

Variable	Component (Group)		
	1	2	3
Cloud coverage (octants)	−0.814		
Relative humidity (%)	−0.900		
% longest sunshine duration (%)	0.890		
Sunshine duration (0.1 h)	0.919		
Global radiation (J/cm^2^)	0.812	0.425	
Minimum temperature (°C)		0.977	
Mean temperature (°C)		0.974	
Maximum temperature (°C)		0.927	
Wind speed (m/s)			0.921
Precipitation (mm)			0.609

## References

[1] Meissner H.C. (2016). Viral bronchiolitis in Children. N. Engl. J. Med..

[2] Shi T., McAllister D.A., O’Brien K.L., Simoes E.A.F., Madhi S.A., Gessner B.D., Polack F.P., Balsells E., Acacio S., Aguayo C. (2017). Global, regional, and national disease burden estimates of acute lower respiratory infections due to respiratory syncytial virus in young children in 2015: A systematic review and modelling study. Lancet.

[3] Fujiogi M., Goto T., Yasunaga H., Fujishiro J., Mansbach J.M., Camargo C.A., Hasegawa K. (2019). Trends in bronchiolitis hospitalizations in the United States: 2000–2016. Pediatrics.

[4] Schlapbach L.J., Straney L., Gelbart B., Alexander J., Franklin D., Beca J., Whitty J.A., Ganu S., Wilkins B., Slater A. (2017). Burden of disease and change in practice in critically ill infants with bronchiolitis. Eur. Respir. J..

[5] Pham H., Thompson J., Wurzel D., Duke T. (2020). Ten years of severe respiratory syncytial virus infections in a tertiary paediatric intensive care unit. J. Paediatr. Child. Health.

[6] Linssen R.S., Bem R.A., Kapitein B., Oude Rengerink K., Otten M.H., den Hollander B., Bont L., van Woensel J.B.M., the PICE Study Group (2021). Burden of respiratory syncytial virus bronchiolitis on the Dutch pediatric intensive care units. Eur. J. Pediatr..

[7] Obando-Pacheco P., Justicia-Grande A.J., Rivero-Calle I., Rodriguez-Tenreiro C., Sly P., Ramilo O., Mejias A., Baraldi E., Papadopoulos N.G., Nair H. (2018). Respiratory syncytial virus seasonality: A global overview. J. Infect. Dis..

[8] Baker R.E., Mahmud A.S., Wagner C.E., Yang W., Pitzer V.E., Viboud C., Vecchi G.A., Metcalf C.J.E., Grenfell B.T. (2019). Epidemic dynamics of respiratory syncytial virus in current and future climates. Nat. Commun.

[9] Yusuf S., Piedimonte G., Auais A., Demmler G., Krishnan S., Van Caeseele P., Singleton R., Broor S., Parveen S., Avendano L. (2007). The relationship of meteorological conditions to the epidemic activity of respiratory syncytial virus. Epidemiol. Infect..

[10] Stensballe L.G., Devasundaram J.K., Simoes E.A. (2003). Respiratory syncytial virus epidemics: The ups and downs of a seasonal virus. Pediatr. Infect. Dis. J..

[11] Meerhoff T.J., Paget J.W., Kimpen J.L., Schellevis F. (2009). Variation of respiratory syncytial virus and the relation with meteorological factors in different winter seasons. Pediatr. Infect. Dis. J..

[12] Sundell N., Andersson L.M., Brittain-Long R., Lindh M., Westin J. (2016). A four year seasonal survey of the relationship between outdoor climate and epidemiology of viral respiratory tract infections in a temperate climate. J. Clin. Virol..

[13] Thongpan I., Vongpunsawad S., Poovorawan Y. (2020). Respiratory syncytial virus infection trend is associated with meteorological factors. Sci. Rep..

[14] Eccles R., Wilkinson J.E. (2015). Exposure to cold and acute upper respiratory tract infection. Rhinology.

[15] Koskela H., Tukiainen H. (1995). Facial cooling, but not nasal breathing of cold air, induces bronchoconstriction: A study in asthmatic and healthy subjects. Eur. Respir. J..

[16] Koskela H.O. (2007). Cold air-provoked respiratory symptoms: The mechanisms and management. Int. J. Circumpolar Health.

[17] Kilgour E., Rankin N., Ryan S., Pack R. (2004). Mucociliary function deteriorates in the clinical range of inspired air temperature and humidity. Intensive Care Med..

[18] Asmundsson T., Kilburn K.H. (1970). Mucociliary clearance rates at various levels in dog lungs. Am. Rev. Respir. Dis..

[19] Mireku N., Wang Y., Ager J., Reddy R.C., Baptist A.P. (2009). Changes in weather and the effects on pediatric asthma exacerbations. Ann. Allergy Asthma Immunol..

[20] Hyrkas H., Ikaheimo T.M., Jaakkola J.J., Jaakkola M.S. (2016). Asthma control and cold weather-related respiratory symptoms. Respir. Med..

[21] (1985). Asthma and the weather. Lancet.

[22] Ferrari U., Exner T., Wanka E.R., Bergemann C., Meyer-Arnek J., Hildenbrand B., Tufman A., Heumann C., Huber R.M., Bittner M. (2012). Influence of air pressure, humidity, solar radiation, temperature, and wind speed on ambulatory visits due to chronic obstructive pulmonary disease in Bavaria, Germany. Int. J. Biometeorol..

[23] Estripeaut D., Torres J.P., Somers C.S., Tagliabue C., Khokhar S., Bhoj V.G., Grube S.M., Wozniakowski A., Gomez A.M., Ramilo O. (2008). Respiratory syncytial virus persistence in the lungs correlates with airway hyperreactivity in the mouse model. J. Infect. Dis..

[24] DeFord D.M., Nosek J.M., Castiglia K.R., Hasik E.F., Franke M.E., Nick B.C., Abdelnour A.M., Haas C.E., Junod N.A., Latsko K.N. (2019). Evaluation of the role of respiratory syncytial virus surface glycoproteins F and G on viral stability and replication: Implications for future vaccine design. J. Gen. Virol..

[25] El Saleeby C.M., Bush A.J., Harrison L.M., Aitken J.A., Devincenzo J.P. (2011). Respiratory syncytial virus load, viral dynamics, and disease severity in previously healthy naturally infected children. J. Infect. Dis..

[26] Colosia A.D., Masaquel A., Hall C.B., Barrett A.M., Mahadevia P.J., Yogev R. (2012). Residential crowding and severe respiratory syncytial virus disease among infants and young children: A systematic literature review. Bmc Infect. Dis..

[27] Chu H.Y., Kuypers J., Renaud C., Wald A., Martin E., Fairchok M., Magaret A., Sarancino M., Englund J.A. (2013). Molecular epidemiology of respiratory syncytial virus transmission in childcare. J. Clin. Virol..

[28] Blanken M.O., Korsten K., Achten N.B., Tamminga S., Nibbelke E.E., Sanders E.A., Smit H.A., Groenwold R.H., Bont L. (2016). Population-attributable risk of risk factors for recurrent wheezing in moderate preterm infants during the first year of life. Paediatr. Perinat. Epidemiol..

[29] Fisman D. (2012). Seasonality of viral infections: Mechanisms and unknowns. Clin. Microbiol. Infect..

[30] Belderbos M.E., Houben M.L., Wilbrink B., Lentjes E., Bloemen E.M., Kimpen J.L., Rovers M., Bont L. (2011). Cord blood vitamin D deficiency is associated with respiratory syncytial virus bronchiolitis. Pediatrics.

[31] Martineau A.R., Jolliffe D.A., Hooper R.L., Greenberg L., Aloia J.F., Bergman P., Dubnov-Raz G., Esposito S., Ganmaa D., Ginde A.A. (2017). Vitamin D supplementation to prevent acute respiratory tract infections: Systematic review and meta-analysis of individual participant data. BMJ.

[32] Hambling M.H. (1964). Survival of the respiratory syncytial virus during storage under various conditions. Br. J. Exp. Pathol..

[33] Paynter S. (2015). Humidity and respiratory virus transmission in tropical and temperate settings. Epidemiol. Infect..

[34] Noyola D.E., Mandeville P.B. (2008). Effect of climatological factors on respiratory syncytial virus epidemics. Epidemiol. Infect..

[35] Walton N.A., Poynton M.R., Gesteland P.H., Maloney C., Staes C., Facelli J.C. (2010). Predicting the start week of respiratory syncytial virus outbreaks using real time weather variables. BMC Med. Inform. Decis. Mak..

[36] Hyrkäs-Palmu H., Ikäheimo T.M., Laatikainen T., Jousilahti P., Jaakkola M.S., Jaakkola J.J.K. (2018). Cold weather increases respiratory symptoms and functional disability especially among patients with asthma and allergic rhinitis. Sci. Rep..

[37] Daviskas E., Gonda I., Anderson S.D. (1990). Mathematical modeling of heat and water transport in human respiratory tract. J. Appl. Physiol..

[38] Freed A.N., Davis M.S. (1999). Hyperventilation with dry air increases airway surface fluid osmolality in canine peripheral airways. Am. J. Respir. Crit. Care Med..

[39] Larsson K., Tornling G., Gavhed D., Müller-Suur C., Palmberg L. (1998). Inhalation of cold air increases the number of inflammatory cells in the lungs in healthy subjects. Eur. Respir. J..

[40] Schuit M., Gardner S., Wood S., Bower K., Williams G., Freeburger D., Dabisch P. (2020). The Influence of Simulated Sunlight on the Inactivation of Influenza Virus in Aerosols. J. Infect. Dis..

[41] Fodha I., Vabret A., Ghedira L., Seboui H., Chouchane S., Dewar J., Gueddiche N., Trabelsi A., Boujaafar N., Freymuth F. (2007). Respiratory syncytial virus infections in hospitalized infants: Association between viral load, virus subgroup, and disease severity. J. Med. Virol..

[42] Sheeran P., Jafri H., Carubelli C., Saavedra J., Johnson C., Krisher K., Sánchez P.J., Ramilo O. (1999). Elevated cytokine concentrations in the nasopharyngeal and tracheal secretions of children with respiratory syncytial virus disease. Pediatr. Infect. Dis. J..

[43] Houben M.L., Coenjaerts F.E., Rossen J.W., Belderbos M.E., Hofland R.W., Kimpen J.L., Bont L. (2010). Disease severity and viral load are correlated in infants with primary respiratory syncytial virus infection in the community. J. Med. Virol..

[44] Scagnolari C., Midulla F., Selvaggi C., Monteleone K., Bonci E., Papoff P., Cangiano G., Di Marco P., Moretti C., Pierangeli A. (2012). Evaluation of viral load in infants hospitalized with bronchiolitis caused by respiratory syncytial virus. Med. Microbiol. Immunol..

[45] Hasegawa K., Jartti T., Mansbach J.M., Laham F.R., Jewell A.M., Espinola J.A., Piedra P.A., Camargo C.A. (2015). Respiratory syncytial virus genomic load and disease severity among children hospitalized with bronchiolitis: Multicenter cohort studies in the United States and Finland. J. Infect. Dis..

[46] Monto A.S. (2008). Epidemiology of influenza. Vaccine.

[47] Lowen A.C., Mubareka S., Steel J., Palese P. (2007). Influenza virus transmission is dependent on relative humidity and temperature. PLoS Pathog..

[48] (2006). Diagnosis and management of bronchiolitis. Pediatrics.

